# Expanding whole exome resequencing into non-human primates

**DOI:** 10.1186/gb-2011-12-9-r87

**Published:** 2011-09-14

**Authors:** Eric J Vallender

**Affiliations:** 1 New England Primate Research Center, Harvard Medical School, One Pine Hill Drive, Southborough, MA 01772, USA

## Abstract

**Background:**

Complete exome resequencing has the power to greatly expand our understanding of non-human primate genomes. This includes both a better appreciation of the variation that exists in non-human primate model species, but also an improved annotation of their genomes. By developing an understanding of the variation between individuals, non-human primate models of human disease can be better developed. This effort is hindered largely by the lack of comprehensive information on specific non-human primate genetic variation and the costs of generating these data. If the tools that have been developed in humans for complete exome resequencing can be applied to closely related non-human primate species, then these difficulties can be circumvented.

**Results:**

Using a human whole exome enrichment technique, chimpanzee and rhesus macaque samples were captured alongside a human sample and sequenced using standard next-generation methodologies. The results from the three species were then compared for efficacy. The chimpanzee sample showed similar coverage levels and distributions following exome capture based on the human genome as the human sample. The rhesus macaque sample showed significant coverage in protein-coding sequence but significantly less in untranslated regions. Both chimpanzee and rhesus macaque showed significant numbers of frameshift mutations compared to self-genomes and suggest a need for further annotation.

**Conclusions:**

Current whole exome resequencing technologies can successfully be used to identify coding-region variation in non-human primates extending into old world monkeys. In addition to identifying variation, whole exome resequencing can aid in better annotation of non-human primate genomes.

## Background

The role of genetic variation in establishing individual differences is well-established. HapMap [1], the Human Genome Diversity Project [2], and most recently the 1,000 Genomes project [3] have all sought to catalog and classify human variation between populations. Human genetic variation is understood to underlie many diseases and exploited to map genetic causes. In model organisms, genetic variation between rodent strains has been commonly used for quantitative trait loci mapping [4]. More recently, the genetic variation between dog breeds has been used to map the genes associated with phenotypic traits [5]. Yet these approaches remain underutilized with regard to non-human primates. A large reason for this is the costs that had been associated with elucidation of polymorphism. The historical importance of rodents in biomedical research coupled with the clonal nature of the strains allowed for significant meaningful genetic data to be gathered from a relatively small population. The relatively lesser importance of the canine model in biomedical research was overcome more recently by lower sequencing costs and again an ability to focus on breeds as 'type-specimens'.

As biomedical research moves into the post-genomic era it is clear that genetic variation in model organisms will only gain in importance. A genomic understanding of variation has led to a re-emergence of the canine model [6]. The importance of genetic variation in non-human primates is beginning to be realized also, particularly in models of infectious disease and behavioral disorders. Genetic variation in the rhesus macaque has been shown to affect viral replication in an HIV model [7,8] and to affect susceptibility to malarial parasites [9]. In studies of behavioral disorders and drug addiction, genetic variation in rhesus macaques has been identified that explains between-individual variance in alcohol consumption [10] and a corresponding response to treatment [11,12], while genetic variation at the tumor necrosis factor promoter region has been identified in vervet monkey models of obesity [13]. Studies such as these not only offer the hope of elucidating the genetic factors underlying human disease, but also are important in the development of truly translational models. Just as animal models of obesity or alcoholism are most valid if their molecular etiologies parallel the underlying human causes, variation affecting the response to pharmaceutical treatment or vaccine efficacy must be appreciated to make sense of study results. So far, however, these studies of polymorphism in non-human primates have remained focused on specific candidate genes.

Our ability to incorporate genetic information into our animal studies is not at issue; rather, the limiting factor has been the difficulty of obtaining genetic data. Resequencing of individual loci has been possible but can be costly. Recently, new technologies, such as complete exome resequencing, have emerged that promise to greatly expand our ability to quickly and practically identify large amounts of polymorphism. As has generally been the case with genomic technologies, exome resequencing began with human studies [14]. Studies in human have already been able to leverage this relatively inexpensive technology to identify novel allele variants associated with disease that have previously eluded researchers [15-17] and it has quickly been applied to numerous diseases and promises to help elucidate many more. This method has already been extended to the Neandertal [18], and if it can be applied to non-human primates, this same technology may offer the opportunity to recapitulate a 'Primate HapMap' at a significantly reduced cost and on a relatively short time scale.

A side benefit to a survey of polymorphism in a species is a much greater depth of genomic coverage for that region. In humans this advantage has been relatively subtle. Because of the primacy and importance of the human genome and institutional focus on it, it is very high quality; resequencing surveys show differences between individuals and populations but generally do not impact our basic understanding and interpretation of the genome. Non-human primate genomes, on the other hand, have been sequenced to a much lower depth of coverage and generally have been subjected to reduced scrutiny. It has been established that there is an apparent excess of pseudogenes in the chimpanzee genome [19,20] and that annotation errors abound [19,21]. As depth of coverage increases these errors will be ameliorated. While ideally this would entail a complete resequencing of the entire genome, much of the most important parts of the genome, certainly those that we currently best understand, can be sequenced at depth using a complete exome approach. It is noteworthy that these comparative approaches have also been successful in improving annotation of the dog genome [22].

Exomic resequencing of non-human primates thus offers significant advantages. The existing non-human primate genomes can be better understood and annotated and tools can be developed that will allow for the incorporation of genetic variation into our primate models of human disease. These in turn allow for a greater translational efficacy and a more refined use of non-human primate animal models. Here we take the first steps towards realizing the promise of this approach, demonstrating its feasibility using existing resources and defining the parameters in which it can be successful. These studies also shed light on the existing non-human primate genomes and offer the beginnings of efforts to refine them.

## Results and discussion

### Exomic coverage following enrichment

The SureSelect Human All Exon Kit, 38 Mb, from Agilent Technologies was used for target enrichment of a male human (*Homo sapiens*), chimpanzee (*Pan troglodytes*), and rhesus macaque (*Macaca mulatta*). The 38 Mb SureSelect kit was designed on the hg18 human genome and included the purported complete human exome as deduced from the NCBI Consensus CDS database as well as an assortment of microRNAs and non-coding RNAs. Human DNA was from a Mbuti pygmy, chosen to capture maximum within-species diversity from the human genome and presumably the SureSelect probes. The chimpanzee and rhesus macaque (of Indian descent) represented individuals unrelated to those used in the assembly of the genomes of their respective species. The enriched exomes were then sequenced on an Illumina GAII using one lane each with a 72-bp paired-end protocol.

In order to assess the overall completeness of the exome capture, each species read was aligned to the human genome (Table 1). Read depth for each species was consistent, with over 60% of targeted regions having over 20 sample reads. For human and chimpanzee, 95% of regions had over 5 sample reads, while for rhesus macaque 95% of regions had more than 2 reads. As expected, high exonic coverage, defined by confidently mapped sample reads to the entirety of the exon, was observed for human while decreasing slightly for chimpanzee and more considerably for rhesus macaque. However, when analysis was restricted to protein-coding regions of the exome only, excluding untranslated regions, the rhesus coverage improved dramatically and both human and chimpanzee coverage incrementally improved (Table 1; Additional file 1). Given that untranslated regions are known to be more divergent between species than protein-coding regions and that the enrichment system operates on homology, this observation is expected. Further, when the coding exons were transliterated to the chimpanzee and rhesus genomes and the sample reads aligned with self-genomes, all species showed approximately 95% of the exome with complete coverage (Table 1), though it must be noted that for both the chimpanzee and rhesus macaque, species-specific true exons would be lost as would legitimate exons for which current genomic sequence is unavailable.

**Table 1:** Sample read coverage

	Human		Chimpanzee		Rhesus macaque	
**Coding exons (human)**						
Complete	150,776	91.9%	148,109	90.3%	105,127	64.1%
Partial	10,583	6.5%	12,776	7.8%	52,941	32.3%
Nothing	2,683	1.6%	3,157	1.9%	5,974	3.6%
All	164,042		164,042		164,042	
						
**CDS (human)**						
Complete	155,669	94.8%	154,322	94.0%	136,123	82.8%
Partial	5,775	3.6%	6,583	4.1%	21,547	13.2%
Nothing	2,598	1.6%	3,137	1.9%	6,372	3.9%
All	164,042		164,042		164,042	
						
**CDS (self-species)**						
Complete	155,669	94.8%	149,541	95.3%	146,606	94.7%
Partial	5,775	3.6%	6,034	3.8%	6,785	4.4%
Nothing	2,598	1.6%	1,293	0.8%	1,469	0.9%
All	164,042		156,868		154,860	

Exomic coverage on the human genome in exon entirety, coding sequence (CDS) only and coding sequence in self-genomes.

Using the self-self alignments, coverage was compared to chromosomal location (Additional file 2). Across all three species a pattern emerged wherein the Y chromosome showed significant failures. The X chromosome also showed a greater percentage of exons without coverage than any autosome, though the difference was much less marked. Three factors appeared to have contributed to these effects, though in different proportions. Firstly, divergence between species is different between the sex chromosomes and autosomes, largely a result of male-driven mutation [23]. Just as untranslated regions showed less coverage, the Y chromosome should be less likely to work in cross-species homology-based approaches. This, however, does not account for the X chromosome nor the significant failure of the approach in the human sample reads. Rather, the main problem plaguing the Y chromosome comes from its repetitive nature, with pseudogenes and closely related gene families abounding [24]. This in turn results in difficulty in assigning reads unambiguously to appropriate positions, a problem in all Y chromosome sequencing efforts. The final effect driving the Y chromosome difficulties and the main effect driving in the X chromosome lack of coverage is simply the smaller effective coverage levels. Each of the autosomes offer twice the starting material as the sex chromosomes and sequencing was not sufficient to achieve saturation.

### Effects of divergence on coverage

**Figure 1:**
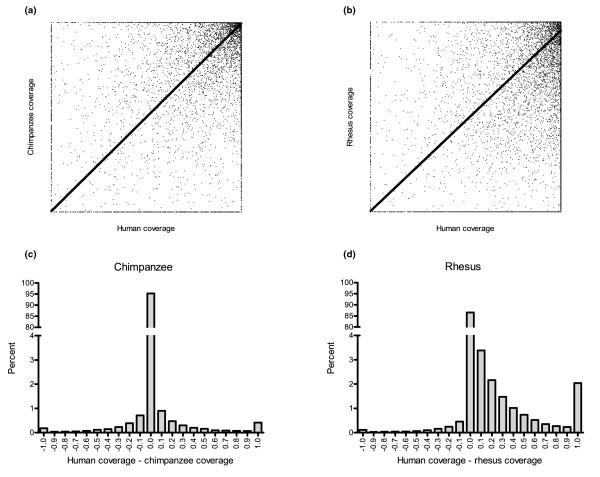
**Human coverage compared to cross-species coverage**. **(a,b) **Scatter plots showing the coverage level for each coding exon from human sample reads on human genome annotation compared to chimpanzee sample reads on human genome annotation (a) or rhesus sample reads on human genome annotation (b). **(c,d) **Histograms showing the difference between human and cross-species coverage, chimpanzee (c) or rhesus macaque (d), demonstrating the effects of species bias in capture efficacy.

In addition to the differences in coverage in the untranslated regions compared to protein-coding regions or in the Y chromosome compared to autosomes, divergence may also play a more general role in the ability of hybridization-based target enrichment approaches to extend across species. For each exon the coverage in human was plotted against the coverage of chimpanzee or rhesus macaque sample reads against the human genome (Figure 1). By treating the chimpanzee and rhesus macaque sample reads simply as extremely divergent but representative of the same genomes, it allowed for a visualization of the effects of divergence on relative levels of coverage. In comparing the chimpanzee to the human it is apparent that there is very little systematic bias in species coverage; almost as many exons show greater coverage in the chimpanzee as in human and at similar levels (Figure 1a,c). In essence, the lack of coverage observed in chimpanzee was no greater than that seen in humans. Coverage in both human and chimpanzee are instead almost entirely bounded by read depth. The rhesus macaque on the other hand shows a loss of coverage due to divergence in addition to that resulting from read depth (Figure 1b,d). Unlike the chimpanzee, the vast majority of exons showing a difference in coverage between the rhesus and human sample reads show a bias towards rhesus deficits. This suggests that divergence levels between rhesus and human are beginning to approach the limits for cross-species hybridization.

**Figure 2:**
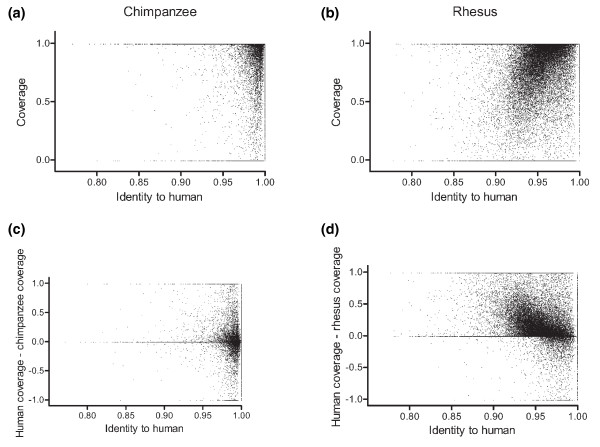
**Coverage compared to divergence**. **(a,b) **Scatter plots showing the relationship between coverage and divergence from human for each coding exon in chimpanzee (a) and rhesus macaque (b). **(c,d) **Scatter plots also show the relationship between divergence from human and coverage differences between human and chimpanzee (c) and rhesus macaque (d).

This becomes clearer when coverage levels are plotted against exonic identity to human (Figure 2). In the chimpanzee, it is evident that there is little to no correlation between divergence and coverage (Figure 2a,c). The coverage levels are dominated by stochastic processes at the levels of nucleotide identity (largely greater than 97%) seen between chimpanzee and human. In rhesus, however, a clear trend is observed (Figure 2b,d). The greater the levels of divergence, the less likely that coverage was observed. As divergence levels become greater than 5% (identity less than 95%), coverage levels begin to fall off fairly rapidly. It should be noted, however, that even at these levels there remain significant numbers of exons that show complete coverage. Species with greater divergence, notably new world monkeys, are likely to suffer significantly while the other ape species are likely to show near complete coverage.

Coverage was also compared using other metrics, including exon length, percent coding, and GC content. None of these factors appeared to play a role in species-specific coverage rates (data not shown). While not observed in these data sets, it does not seem unlikely that, in situations of greater divergence, one or more of these factors may play a major role. It is important to note that the findings here are confined to an exomic capture strategy; they are not necessarily directly applicable to other regions. Cross-species capture of regions of low complexity including, for example, repeats or CpG islands, are likely to be more greatly influenced by these factors.

### Identification and comparison of within-species variation

The primary goal of whole exome resequencing is the identification of polymorphism. The success of this approach in humans is beginning to be felt already. At the same time, it will be particularly useful in outbred model organisms, especially non-human primates. This basic conceit motivated these studies. Using the self-self genomic alignments, it was possible to identify variation in the individuals compared to the reference genomes (Table 2). For the most part, results were as expected and painted a picture of a successful approach. Total numbers of synonymous and non-synonymous SNPs were consistent with previous estimates. The larger levels of polymorphism observed in rhesus macaques is consistent with a larger effective population size. Similarly, ratios of non-synonymous to synonymous polymorphism and rates of pseudogenization via nonsense mutations are roughly consistent with expected values accounting for the effects of selection and genetic drift. Notable here in particular is the ratio of heterozygous nonsense mutations to homozygous mutations, less than 5% in human and chimpanzee and 10% in rhesus macaque. This represents, of course, not just standard expectations of genotypic frequency patterns, but also a likely deleterious effect of homozygous pseudogenization in many genes.

**Table 2:** Nucleotide variation in self-self assemblies

	Human			Chimpanzee			Rhesus macaque		
			
	Hom	Het	Hemi	Hom	Het	Hemi	Hom	Het	Hemi
**Deletions**									
Frameshift	10	53	0	114	119	15	169	162	4
Amino acid loss	13	63	0	7	68	3	16	73	0
									
**Insertions**									
Frameshift	13	52	4	85	83	22	128	169	6
Amino acid gain	12	42	1	5	57	1	9	62	1
									
**MNP**									
Synonymous	23	34	2	22	57	5	20	77	1
Non-synonymous	79	269	2	312	571	47	245	793	9
Stop codon gain	0	7	0	1	19	2	0	25	1
Stop codon loss	1	0	0	0	2	0	0	1	0
									
**SNP**									
Synonymous	4,259	8,159	206	3,204	11,549	198	8,777	14,878	233
Non-synonymous	3,618	8,336	155	3,725	12,465	346	6,158	13,252	220
Stop codon gain	8	171	2	11	286	2	34	339	3
Stop codon loss	9	7	0	2	21	0	18	20	1
									
**Genes**									
Deletion frameshift	10		0	107		14	159		4
Insertion frameshift	13		4	85		21	125		6
Either	23		4	182		35	280		9
									
Stop codon gain	8		2	12		4	34		4
Stop codon loss	10		0	2		0	17		1
Either	18		2	14		4	51		5
									
Any of above	41		6	194		38	327		14

Nucleotide level variation observed in self-self assemblies. Hemi, hemizygous; Het, heterozygous; Hom, homozygous; MNP, multi-nucleotide polymorphism; SNP, single-nucleotide polymorphism.

These conventionally expected results are in contrast to frameshift mutations following an insertion or deletion. The number of human frameshift mutations and their ratio of homozygosity to heterozygosity, while higher than that seen in nonsense mutations, are still generally comparable. This is confirmed when insertions and deletions in multiples of three, resulting in the gain or loss of amino acids but not frameshifts, are considered. In both chimpanzee and rhesus macaque, however, we see approximately equal numbers of homozygous and heterozygous frameshifts. This is in contrast to the amino acid gain and loss numbers, which remain similar to the human values. These data suggest an excess of homozygous frameshift mutations in chimpanzee and rhesus macaque.

The most straightforward explanation for this is that these frameshifts do not occur in isolation and that their action in combination 'corrects' the gene. This could arise either biologically or, more likely, as the result of local misalignments. To assess this, frameshift mutations, as well as stop gains and losses from SNPs, were gathered into genes. While there are some examples of these appearing in combination, by and large these are unique events that do not appear 'corrected' in their genomes. While biological formally possible, a more parsimonious explanation for these large differences may be errors in the genome or otherwise poor or incomplete annotations.

### Inferred divergence between species and comparison to existing genomes

The human genome is, naturally, the most complete and high quality, in terms of both sequence confidence and annotation, of the mammalian genomes. In order to test whether the frameshifts observed when the chimpanzee and rhesus sample reads were aligned against self-genomes were truly biologically representative or artifactual results from genomic deficiencies, the chimpanzee and rhesus macaque next generation sample reads were aligned to the human genome (hg18). Also faux next generation sequencing (NGS) reads were created from the chimpanzee (panTro2) and rhesus (rheMac2) genome assemblies and aligned to the human genome. A summary of the observed nucleotide level variation can be found in Table 3.

**Table 3:** Nucleotide variation in assemblies to human

	Chimpanzee						Rhesus macaque					
		
	Sample			Genome			Sample			Genome		
				
	Hom	Het	Hemi	Hom	Het*	Hemi	Hom	Het	Hemi	Hom	Het*	Hemi
**Deletions**												
Frameshift	147	185	11	550	296	72	337	360	18	504	200	32
Amino acid loss	191	210	12	183	74	10	643	295	35	697	290	37
												
**Insertions**												
Frameshift	104	163	11	242	260	29	190	321	15	281	300	17
Amino acid gain	109	269	13	69	237	3	319	600	27	246	755	34
												
**MNP**												
Synonymous	289	125	10	78	13	4	1,310	318	81	963	52	41
Non-synonymous	1,986	1,024	56	1,971	293	79	13,022	2,822	502	17,762	982	682
Stop codon gain	7	27	1	20	16	4	36	106	1	55	28	0
Stop codon loss	2	2	0	0	2	0	8	4	0	6	2	0
												
**SNP**												
Synonymous	76,204	14,506	2,355	75,930	826	1,864	318,662	23,085	11,117	346,779	2,688	11,653
Non-synonymous	49,699	16,017	2,220	49,093	1,105	1,696	139,476	22,633	5,943	160,418	2,574	6,479
Stop codon gain	204	379	19	228	17	15	400	732	20	445	26	21
Stop codon loss	85	34	4	27	1	3	137	50	5	75	2	3
												
**Genes**												
Deletion frameshift	143		10	489 (678)^a^		55	328		17	438 (579)^a^		27
Insertion frameshift	101		10	230 (439)^a^		28	180		14	266 (519)^a^		16
Either	241		20	679 (1,016)^a^		76	489		29	665 (1,013)^a^		39
												
Stop codon gain	200		16	237 (259)^a^		14	406		21	443 (494)^a^		19
Stop codon loss	87		4	28 (32)^a^		0	143		5	80 (84)^a^		3
Either	280		19	263 (289)^a^		14	534		26	513 (567)^a^		22
												
Any of above	499		39	895 (1,316)^a^		87	945		51	1082 (1,425)^a^		56

Comparison of nucleotide level variation in sample reads and genomic cross-species alignments to human. ^a^Values in parentheses are Hom + Het*. Hemi, hemizygous; Het, heterozygous; Hom, homozygous; MNP, multi-nucleotide polymorphism; SNP, single-nucleotide polymorphism.

The first, and most obvious, observation from these data is that there remain some issues in assembly. The chimpanzee and rhesus faux-NGS reads from genomes are effectively haploid and yet autosomal 'heterozygous' mutations exist. Notable here is that these assembly errors are heavily biased towards insertions/deletions, where they represent nearly 50% of the total insertion/deletion events, compared to SNP or multi-nucleotide polymorphism (MNP) events, where they represent less than 1.5%. The effect of these 'heterozygous' variations, however, does not alter the most important finding, but rather just suggests that, if anything, it is conservative.

That primary finding is that the chimpanzee and rhesus genomes still contain numerous incorrect insertion/deletion differences. Comparing top-line data, the chimpanzee sample reads showed 114 homozygous frameshift deletions and 85 homozygous frameshift insertions when aligned to the chimpanzee genome. When aligned to the human genome these numbers were remarkably similar, 147 and 104, respectively. The most parsimonious explanation would hold that the differences between the sample reads and each of the two genomes largely overlap and represent mildly deleterious mutations, part of this individual's genetic load. However, when the chimpanzee genomic sequence is aligned to the human genomic sequence the corresponding values are 550 and 242 and when the variants are compared there is little overlap.

What seems to be happening is that when the chimpanzee sample reads are aligned to the human genome, more-or-less 'real' insertion/deletion events are being identified. These include both polymorphisms unique to the specific chimpanzee sequenced as well as true divergence events between the species. However, most of the differences between the chimpanzee sequence reads and the chimpanzee genome, rather than representing true polymorphisms like SNP and MNP variation, though undoubtedly some of these do exist, instead represent errors in genomic annotation. These two sources of error - true frameshift mutational events and errors in chimpanzee genomic annotation - are combined in the comparison between the chimpanzee genome and the human genome, though the numbers are slightly higher due to incomplete coverage in the chimpanzee sequence reads.

Applying a similar analysis to the rhesus macaque generates similar findings, though to a lesser degree. There appear to be more true homozygous insertion and deletion frameshifts in rhesus compared to human - 337 and 190, respectively - but this is unexpected given the phylogenetic relationship between the three species. The number of apparently false insertion/deletion frameshifts - 169 and 128, respectively - are roughly similar to that seen in chimpanzee. Further, the total differences observed between the rhesus genome and the human genome, 504 and 281, are very similar to the total number of differences between the chimpanzee genome and human genome and leave fewer insertion/deletion events unaccounted for. It is perhaps notable as well that we would expect the rhesus genome to have an overall poorer alignment to the human genome - resulting from both biologically 'true' and methodological effects - because of the increased divergence. Indeed this is what we observe in the initial coverage comparisons.

Two other observations from these data are worth noting. First, this pattern does not appear to extend to stop codon gains or losses resulting from SNPs or MNPs. Most observed differences between the chimpanzee or rhesus macaque genomes and the human genomes are also observed in the newly sequenced samples. Second, these errors are not insignificant. In the rhesus macaque seemingly false frameshifts affect 200 genes; in chimpanzee this may be the lower bound, with perhaps another 200 frameshifted genes between the chimpanzee and human genomes unaccounted for.

## Conclusions

As initially conceived, this project focused on the relative utility of human complete exome capture technologies to determine variation in protein coding regions within non-human primate populations. In this it is largely successful. Coverage of the chimpanzee is nearly identical to that seen in humans, with no species-specific bias seemingly occurring. With the exception of chimpanzee-specific duplications, it can be reasonably asserted that complete coverage can be gained by these approaches. For rhesus macaque, the percent coverage declines somewhat, but nevertheless coverage between 80 and 95% should be reasonable. As expected, efficacy is directly correlated with divergence and those regions showing least conservation between species are the least likely to be covered.

Based on the correlation between divergence and coverage, the cutoff for capture without bias, as seen in the chimpanzee, seems to be around 96% similarity. From this we should be able to interpolate that this method will be equally efficacious for gorilla and orangutan as it is for chimpanzee. The lesser apes, gibbons and siamangs, will show some loss of coverage and may be strongly affected by the major genomic reorganization events that appear to have taken place within the lineage [25]. While most old world monkeys, notably baboons (*Papio *sp.) and vervet monkeys (*Chlorocebus aethiops*), should show coverage similar to rhesus macaques, new world monkeys likely will not be particularly amenable to this approach save for particularly highly conserved regions. Nevertheless, these results suggest that the development of these methods can be applied to future studies aimed at cataloging variation in numerous biomedically important non-human primate species.

At the same time, an important secondary use of these data is to validate and deepen our current non-human primate genomes. On this front, it has also proven extremely useful. Anecdotal evidence has suggested that there are errors in the chimpanzee and rhesus macaque genomes resulting in poor or incorrect annotations. Most notably this has caused many genes in the chimpanzee and rhesus genomes to be annotated as pseudogenes when they are fully intact and assumedly functional. Here we are able to identify significant numbers of situations in which the chimpanzee or rhesus sample reads look like the human genome while the chimpanzee and rhesus genomes harbor a frameshift.

The two samples presented here, one chimpanzee and one rhesus macaque, by themselves are not going to fix the annotation of their species genomes; rather, they serve only as an initial suggestion that not all may be well. Falsely identified polymorphisms will require many more individuals to be conclusively called. In fact, there is little evidence contained in this study that there is any pervasive difference. It is also important to note that many of the worst offenders in annotation problems are the result of the addition of exons to genes that are not present in humans. While the resequencing of the human exome in another species may add exonic sequences that are currently absent from other genomes, it will not comment on the validity of these newly introduced exons. Indeed, while this approach will generally be useful for conserved genes, those with recent paralogs will be missed entirely.

Yet despite its limitations, it is important to recognize the utility of this approach. This methodology allows for the rapid and relatively inexpensive capture of significant amounts of genetic information, both for species with known genomes as well as for other, closely related species without complete sequence. It allows for the identification of polymorphism in rhesus macaques that can be used to refine their use in translational studies and to approach non-human primate genetic modeling of human disease in a unique fashion. Finally, it begins to further our understandings of the chimpanzee and rhesus macaque genomes and will easily add depth of coverage to the coding regions in the genomes, work that can be easily extended to the impending gorilla, orangutan, baboon, and vervet monkey genomes.

Whole exome resequencing is an important new tool in the geneticist's arsenal and one that is not reserved for human genetic work. Indeed, where it is likely to see some of its greatest utility is in species for which polymorphism has been largely overlooked. The fact that tools developed for humans can be applied with reasonable confidence to non-human primates augurs well for these species and their development as true genetic translational models.

## Materials and methods

### Genomic DNA samples

Human (NA10495) and chimpanzee (NS03641) genomic DNA was obtained from the Coriell Cell Repository. The human DNA, line JK1033, was from an adult male Mbuti pygmy living in the Ituri forest of northern Zaire made available through the NIGMS Human Genetics Cell Repository. The chimpanzee DNA was from 'Juan', a 32 year old male housed at the Yerkes National Primate Research Center. Genomic DNA from an adult male rhesus macaque of Indian descent housed at the New England National Primate Research Center was obtained from the NEPRC Primate Genetics Core [26]. In brief, approximately 8 ml of venous blood was collected in and EDTA-Vacutainer tube during a routine physical. Genomic DNA was then isolated and purified using the Flexigene kit (Qiagen, Valencia, CA, USA).

### Exome capture and sequencing

The SureSelect Human All Exon Kit, 38 Mb (Agilent Technologies, Santa Clara, CA, USA) was used to capture the exomes from each of the three species using the manufacturer's protocols; 10 μg of genomic DNA from each species was used. Library preparation was performed using the NEBNext Sample Preparation Kit (New England Biolabs, Ipswich, MA, USA) using primer and adaptor oligonucleotides from Illumina. Samples were quality control tested using the Agilent 2100 Bioanalyzer and SYBR Green-based quantitative PCR assays. All samples were sequenced on an Illumina Genome Analyzer II using a 72-bp paired-read protocol. Exome capture, library preparation and next generation sequencing were performed according to the manufacturer's protocols in the Biopolymers Facility, Department of Genetics, at Harvard Medical School. Sequence reads have been submitted to the NCBI Sequence Read Archive (SRA038332).

### Data analysis

Initial data analysis, including alignment to genome, coverage analysis, and nucleotide-level variation analysis, used DNAnexus (Palo Alto, CA, USA). Sequencing reads from all three species were aligned to the human genome build hg18/NCBI36.1. Track files containing the genomic regions enriched in the SureSelect Human All Exon Kit, 38 Mb were provided by Agilent Technologies. Chimpanzee sequencing reads were also mapped to the CGSC2.1/panTro2 chimpanzee genome assembly. Rhesus macaque sequencing reads were also mapped to the MGSC1.0/rheMac2 rhesus genome assembly. The genomic exome regions from human (hg18) were converted to chimpanzee (panTro2) and rhesus (rheMac2) using the liftOver program available from the UCSC [27]. Faux-NGS reads were generated using a 72-bp sliding window with a 1-bp step. The faux-NGS chimpanzee and rhesus genomes were then aligned to the human genome (hg18) in the same manner as the true NGS reads. Separately, the Bowtie package [28] was also used to align sample reads to genomes without significantly different results.

## Abbreviations

bp: base pair; MNP: multi-nucleotide polymorphism; NGS: next generation sequencing; SNP: single-nucleotide polymorphism.

## Authors' contributions

EJV executed the study and wrote the manuscript.

## Supplementary Material

Additional file 1 **Additional Figure 1 - exonic coverage across species**. Percent coverage of coding exons are binned and presented as a histogram. **(a-c) **Coverage percentages are based on alignments between human (a), chimpanzee (b), and rhesus macaque (c) and the human genome (hg18) with gene sequences defined by RefSeq annotations. Click here for file

Additional file 2 **Additional Figure 2 - chromosomal distribution of coverage failure**. Percent of coding exons without any coverage by chromosomal position. Y chromosome exons are consistently and substantially more likely to show no coverage compared to autosomal exons. X chromosome exons are also more likely to show no coverage though to a lesser extent. Both trends hold across species. Click here for file

## References

[B1] HapMap Consortium I A haplotype map of the human genome. Nature 2005 437 1299 1320 10.1038/nature04226 16255080 PMC1880871

[B2] Cann HM de Toma C Cazes L Legrand MF Morel V Piouffre L Bodmer J Bodmer WF Bonne-Tamir B Cambon-Thomsen A Chen Z Chu J Carcassi C Contu L Du R Excoffier L Ferrara GB Friedlaender JS Groot H Gurwitz D Jenkins T Herrera RJ Huang X Kidd J Kidd KK Langaney A Lin AA Mehdi SQ Parham P Piazza A Pistillo MP A human genome diversity cell line panel. Science 2002 296 261 262 11954565 10.1126/science.296.5566.261b

[B3] Durbin RM Abecasis GR Altshuler DL Auton A Brooks LD Gibbs RA Hurles ME McVean GA A map of human genome variation from population-scale sequencing. Nature 2010 467 1061 1073 10.1038/nature09534 20981092 PMC3042601

[B4] Klein TW Analysis of major gene effects using recombinant inbred strains and related congenic lines. Behav Genet 1978 8 261 268 10.1007/BF01072828 687318

[B5] Ostrander EA Wayne RK The canine genome. Genome Res 2005 15 1706 1716 10.1101/gr.3736605 16339369

[B6] Parker HG Shearin AL Ostrander EA Man's best friend becomes biology's best in show: genome analyses in the domestic dog. Annu Rev Genet 2010 44 309 336 10.1146/annurev-genet-102808-115200 21047261 PMC3322674

[B7] Kirmaier A Wu F Newman RM Hall LR Morgan JS O'Connor S Marx PA Meythaler M Goldstein S Buckler-White A Kaur A Hirsch VM Johnson WE TRIM5 suppresses cross-species transmission of a primate immunodeficiency virus and selects for emergence of resistant variants in the new species. PLoS Biol 2010 8 e1000462 10.1371/journal.pbio.1000462 20808775 PMC2927514

[B8] Lim SY Rogers T Chan T Whitney JB Kim J Sodroski J Letvin NL TRIM5alpha modulates immunodeficiency virus control in rhesus monkeys. PLoS Pathog 2010 6 e1000738 10.1371/journal.ppat.1000738 20107597 PMC2809762

[B9] Flynn S Satkoski J Lerche N Kanthaswamy S Smith DG Genetic variation at the TNF-alpha promoter and malaria susceptibility in rhesus (*Macaca mulatta*) and long-tailed (*Macaca fascicularis*) macaques. Infect Genet Evol 2009 9 769 777 10.1016/j.meegid.2009.03.011 19570728

[B10] Barr CS Goldman D Non-human primate models of inheritance vulnerability to alcohol use disorders. Addict Biol 2006 11 374 385 10.1111/j.1369-1600.2005.00034.x 16961765

[B11] Barr CS Chen SA Schwandt ML Lindell SG Sun H Suomi SJ Heilig M Suppression of alcohol preference by naltrexone in the rhesus macaque: a critical role of genetic variation at the micro-opioid receptor gene locus. Biol Psychiatry 2010 67 78 80 10.1016/j.biopsych.2009.07.026 19748082 PMC2794913

[B12] Vallender EJ Ruedi-Bettschen D Miller GM Platt DM A pharmacogenetic model of naltrexone-induced attenuation of alcohol consumption in rhesus monkeys. Drug Alcohol Depend 2010 109 252 256 10.1016/j.drugalcdep.2010.01.005 20153935 PMC2875311

[B13] Gray SB Howard TD Langefeld CD Hawkins GA Diallo AF Wagner JD Comparative analyses of single-nucleotide polymorphisms in the TNF promoter region provide further validation for the vervet monkey model of obesity. Comp Med 2009 59 580 588 20034434 PMC2798838

[B14] Albert TJ Molla MN Muzny DM Nazareth L Wheeler D Song X Richmond TA Middle CM Rodesch MJ Packard CJ Weinstock GM Gibbs RA Direct selection of human genomic loci by microarray hybridization. Nat Methods 2007 4 903 905 10.1038/nmeth1111 17934467

[B15] Choi M Scholl UI Ji W Liu T Tikhonova IR Zumbo P Nayir A Bakkaloglu A Ozen S Sanjad S Nelson-Williams C Farhi A Mane S Lifton RP Genetic diagnosis by whole exome capture and massively parallel DNA sequencing. Proc Natl Acad Sci USA 2009 106 19096 19101 10.1073/pnas.0910672106 19861545 PMC2768590

[B16] Ng SB Turner EH Robertson PD Flygare SD Bigham AW Lee C Shaffer T Wong M Bhattacharjee A Eichler EE Bamshad M Nickerson DA Shendure J Targeted capture and massively parallel sequencing of 12 human exomes. Nature 2009 461 272 276 10.1038/nature08250 19684571 PMC2844771

[B17] Ng SB Buckingham KJ Lee C Bigham AW Tabor HK Dent KM Huff CD Shannon PT Jabs EW Nickerson DA Shendure J Bamshad MJ Exome sequencing identifies the cause of a mendelian disorder. Nat Genet 2010 42 30 35 10.1038/ng.499 19915526 PMC2847889

[B18] Burbano HA Hodges E Green RE Briggs AW Krause J Meyer M Good JM Maricic T Johnson PL Xuan Z Rooks M Bhattacharjee A Brizuela L Albert FW de la Rasilla M Fortea J Rosas A Lachmann M Hannon GJ Pääbo S Targeted investigation of the Neandertal genome by array-based sequence capture. Science 2010 328 723 725 10.1126/science.1188046 20448179 PMC3140021

[B19] Wetterbom A Ameur A Feuk L Gyllensten U Cavelier L Identification of novel exons and transcribed regions by chimpanzee transcriptome sequencing. Genome Biol 2010 11 R78 10.1186/gb-2010-11-7-r78 20653958 PMC2926789

[B20] Wetterbom A Gyllensten U Cavelier L Bergstrom TF Genome-wide analysis of chimpanzee genes with premature termination codons. BMC Genomics 2009 10 56 10.1186/1471-2164-10-56 19178713 PMC2640416

[B21] Vallender EJ Bioinformatic approaches to identifying orthologs and assessing evolutionary relationships. Methods 2009 49 50 55 10.1016/j.ymeth.2009.05.010 19467333 PMC2732758

[B22] Derrien T Theze J Vaysse A Andre C Ostrander EA Galibert F Hitte C Revisiting the missing protein-coding gene catalog of the domestic dog. BMC Genomics 2009 10 62 10.1186/1471-2164-10-62 19193219 PMC2644713

[B23] Makova KD Li WH Strong male-driven evolution of DNA sequences in humans and apes. Nature 2002 416 624 626 10.1038/416624a 11948348

[B24] Rozen S Skaletsky H Marszalek JD Minx PJ Cordum HS Waterston RH Wilson RK Page DC Abundant gene conversion between arms of palindromes in human and ape Y chromosomes. Nature 2003 423 873 876 10.1038/nature01723 12815433

[B25] Misceo D Capozzi O Roberto R Dell'oglio MP Rocchi M Stanyon R Archidiacono N Tracking the complex flow of chromosome rearrangements from the Hominoidea ancestor to extant Hylobates and Nomascus gibbons by high-resolution synteny mapping. Genome Res 2008 18 1530 1537 10.1101/gr.078295.108 18552313 PMC2527702

[B26] Ferguson B Capitanio J Folks T Hotchkiss C Johnson Z Kean L Kubisch HM Lank S Lyons L Miller GM Nylander J O'Connor D Vallender EJ Wiseman R Resource brief: the National Non-Human Primate DNA Bank. Methods 2009 49 3 4 10.1016/j.ymeth.2009.07.011 19706346 PMC3172814

[B27] liftOver. http://hgdownload.cse.ucsc.edu/downloads.html#liftover

[B28] Langmead B Trapnell C Pop M Salzberg SL Ultrafast and memory-efficient alignment of short DNA sequences to the human genome. Genome Biol 2009 10 R25 10.1186/gb-2009-10-3-r25 19261174 PMC2690996

