# Supplementary figures and images for: Expanding whole exome resequencing into non-human primates
Source: Genome Biol. 2011 Sep 14;12(9):R87. doi: 10.1186/gb-2011-12-9-r87 (PMC3308050; doi:10.1186/gb-2011-12-9-r87)

### Human

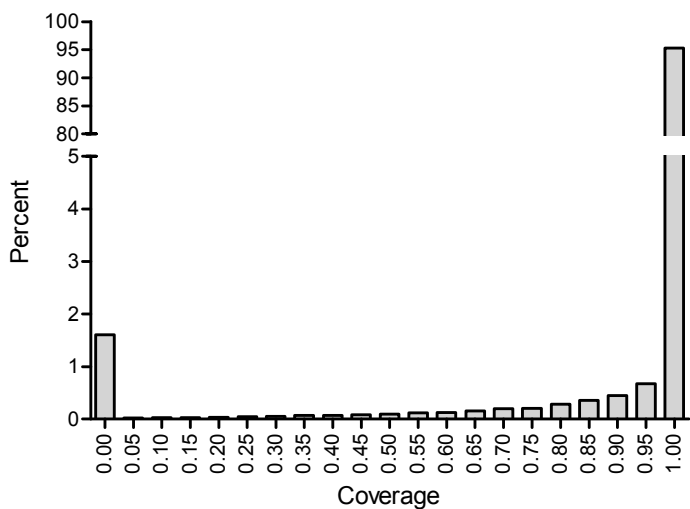

### Chimpanzee

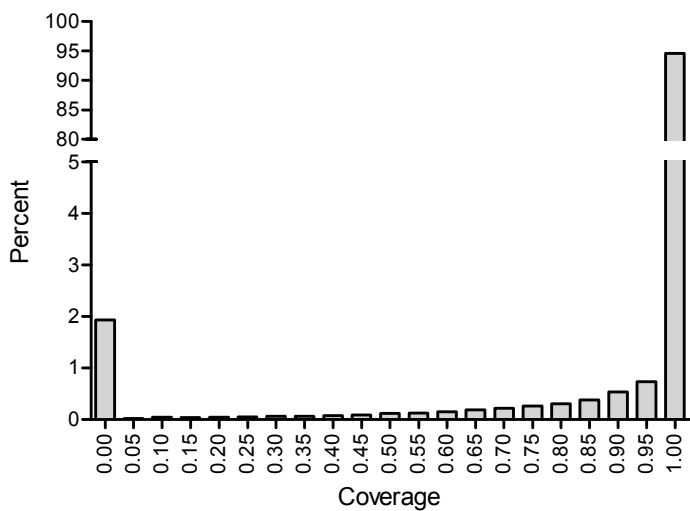

### Rhesus

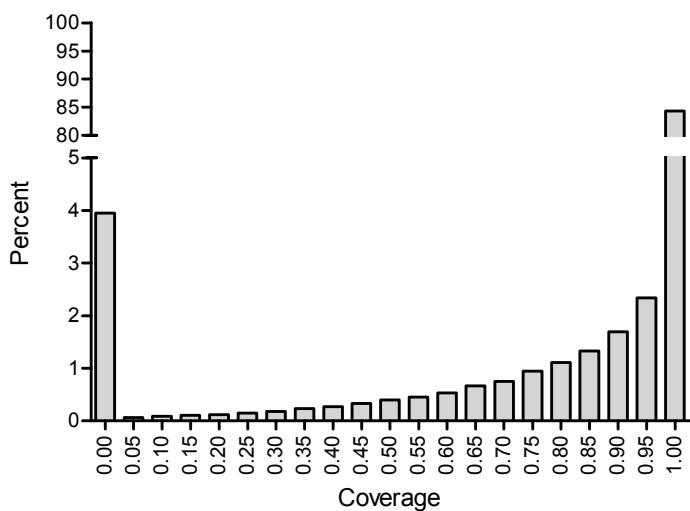

Supplement: Additional file 1 — Additional Figure 1 - exonic coverage across species. Percent coverage of coding exons are binned and presented as a histogram. (a-c) Coverage percentages are based on alignments between human (a), chimpanzee (b), and rhesus macaque (c) and the human genome (hg18) with gene sequences defined by RefSeq annotations. [file gb-2011-12-9-r87-S1.PDF]

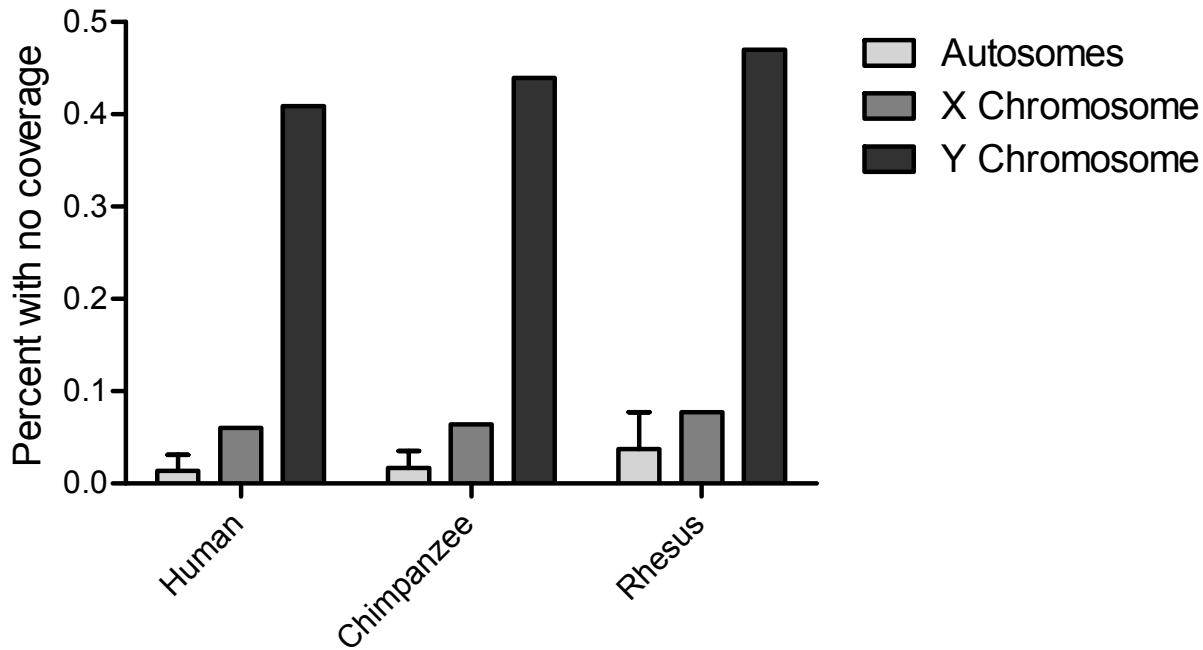

Supplement: Additional file 2 — Additional Figure 2 - chromosomal distribution of coverage failure. Percent of coding exons without any coverage by chromosomal position. Y chromosome exons are consistently and substantially more likely to show no coverage compared to autosomal exons. X chromosome exons are also more likely to show no coverage though to a lesser extent. Both trends hold across species. [file gb-2011-12-9-r87-S2.PDF]
